# Structure-Selective Polydopamine Coating on Drug Nanoparticles

**DOI:** 10.1021/acsami.5c19920

**Published:** 2026-01-26

**Authors:** Danna Niezni, Dana Meron Azagury, Maytal Avrashami, Orr Bar-Natan, Yosi Shamay

**Affiliations:** † Department of Biomedical Engineering 26747 Technion − Israel Institute of Technology, Haifa, 32000, Israel; ‡ The Norman Seiden Multidisciplinary Program for Nanoscience and Nanotechnology, Technion − Israel Institute of Technology, Haifa, 32000, Israel

**Keywords:** polydopamine, nanoparticles, nanoprecipitation, drug delivery, nanomedicine, decision tree

## Abstract

Polydopamine (PDA) is widely regarded as a universal coating material with substrate-independent adhesion. Here we report the first demonstration of selective PDA coating on small molecule drugs, revealing unexpected structure-dependent behavior that challenges this paradigm. Systematic screening of 30 chemotherapeutic agents in IR783-stabilized nanoparticles (>90% drug loading) showed dramatic coating variations governed by molecular structure rather than conventional hydrophobic or π–π interactions. Using Dragon molecular descriptors and principal component analysis, we developed a predictive decision tree model based on nitrogen content and bonding topology that correctly classified 80% of validation compounds. Coating selectivity correlates primarily with nitrogen percentage and N–C–N motifs, fundamentally expanding the understanding of PDA surface chemistry beyond nonselective adhesion mechanisms. Representative drugs (trametinib, dasatinib) demonstrated that PDA coating significantly improves colloidal stability and reduces aggregation without compromising drug loading or release kinetics. *In vivo* evaluation in HCT116 xenografts confirmed superior efficacy over free drug with improved formulation stability. These findings establish PDA coating selectivity as a previously unrecognized material phenomenon and provide computational tools for rational nanoparticle surface design.

## Graphical abstract

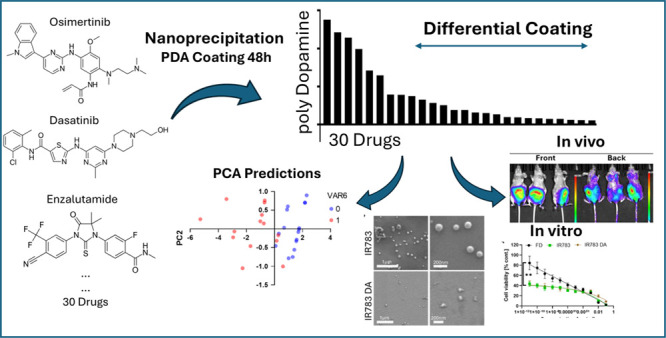

## Introduction

Nanoformulations of small molecule drugs are being developed to effectively deliver treatments for numerous diseases. Current clinically approved systems, including liposomes, lipid nanoparticles, and polymer micelles, typically exhibit drug loading capacities of only 5–15%, limiting their therapeutic efficiency and requiring large doses to reach effective tumor concentrations.^1^ Recently, nanoparticles formed via coassembly of hydrophobic drugs with sulfated organic dyes have gained significant interest, achieving exceptional drug-loading capacities of up to 90–95% while maintaining simple synthesis protocols.^2,3^

These systems have proven to be readily predictable through machine learning approaches and demonstrated efficacy in *in vivo* tumor models via caveolae-mediated transport. However, despite their promise, dye-stabilized nanoparticles face several limitations including lack of active targeting capabilities, limited shelf life, and poor long-term stability.^4,5^ To address these challenges, surface modification strategies represent an underexplored, yet promising avenue. Currently, research on functional coatings for dye-stabilized systems remains limited despite their potential to overcome existing limitations.^6^ We hypothesize that developing functional surface coatings for these high-loading drug delivery systems could significantly improve their biodistribution profiles and formulation stability, thereby expanding the therapeutic applications of dye-based nanoparticles.

Polydopamine (PDA) is a bioinspired polymer derived from dopamine that has rapidly gained importance as a coating material for diverse substrates. It mimics the adhesive proteins found in mussels,^7^ resulting in exceptional surface adhesion and versatility in biomedical and materials science applications.

PDA is structurally related to melanin, an endogenous polymer responsible for skin pigmentation. Its polymerization process is a spontaneous reaction of dopamine oxidation in mild alkaline conditions.^8–10^ The process is robust, cost-effective, and environmentally friendly, requiring neither organic solvents, complex equipment, nor other hazardous chemicals. The resulting polymer is known for its uniformity and stability, providing a reliable platform for further functionalization.^11,12^ The coating ability of PDA arises from its unique chemical structure, which is rich in catechol and amine functional groups. These groups enable strong covalent and noncovalent interactions (such as π–π stacking, hydrogen bonding, and metal chelation) with a wide range of surfaces.^13,14^ Moreover, the process can be easily modified; parameters such as pH, temperature, and reaction time can be adjusted to control the thickness and morphology of the PDA layer.^15^

As a melanin-like material, PDA is inherently biocompatible and biodegradable, making it ideal for biomedical applications such as drug delivery, tissue engineering, and biosensing.^16,17^ PDA coatings help mitigate adverse biological responses, such as inflammation and immune reactions, improving the safety and long-term compatibility of coated materials *in vivo*.^18^ It can also improve biocompatibility,^19^ photothermal properties^20^ and drug release rates.^15^ PDA-coated nanoparticles combine the unique adhesive, chemical, and biological properties of PDA with the functional versatility of nanomaterials, offering a powerful platform for a wide range of scientific and technological applications.^8,16^ PDA coatings have previously been published with various types of nanoparticles, including metallic nanoparticles (e.g., gold)^21^ and polymeric nanoparticles (e.g., PLGA-based).^22^

In this study, we applied a PDA coating to IR783-stabilized nanoprecipitated nanoparticles, which achieve up to 90% drug loading through a straightforward one-pot synthesis.^5,23^ We hypothesized that the PDA coating could expand the range of drugs amenable to a stable formulation while improving nanoparticle stability and performance. Furthermore, PDA’s capacity for surface functionalization enables future conjugation of targeting peptides or bioactive motifs.^24,25^ Unexpectedly, we discovered a differential PDA coating efficiency that varies with the encapsulated drug. Through systematic screening of 30 drugs, we developed a predictive decision tree model and comprehensively characterized the coating phenomenon, revealing that selectivity is governed by specific molecular features and offering new strategies for rational nanoparticle design with coating and further bioconjugation feasible with amines and thiols.

## Materials and Methods

### Materials and Reagents

Nondrug chemicals were purchased from Sigma-Aldrich (St. Louis, MO, USA). Dimethyl sulfoxide (DMSO) was purchased from Carlo Erba (Emmendingen, Germany); sodium bicarbonate was purchased from Bio Lab Chemicals (Jerusalem, Israel). All drugs were purchased from LC-Laboratories (Woburn, MA, USA) and MedChemExpress (Monmouth Junction, NJ, USA).

### Nanoparticle Preparation

Drugs dissolved in DMSO (10 mg/mL) were added under slight vortex to an aqueous IR783 (Sigma-Aldrich) solution (2 mg/mL), buffered with 0.1 M sodium bicarbonate; the process is done without light protection. To form the coating, dopamine monomer dissolved in double-distilled water (DDW,4 mg/mL) was added with a second slight vortex and incubated at room temperature for 48 h. The solution was centrifuged (30,000*g*, 15 min, RT), and the pellet was resuspended in 1 mL of DDW. The pellet resuspension was sonicated using Sonics’ (Newtown, CT, USA) Vibra-cell ultrasonic processor (20% amplitude, 3 s pulses) until homogeneous.

### Absorbance Measurements

Absorbance measurements were evaluated with a Synergy H1 (BioTek, Santa Clara, CA, USA) plate reader. Absorbance was measured at a 490 nm wavelength to evaluate the presence of polydopamine.

### IR783 Degradation by Hydrogen Peroxide

Noncoated nanoparticles were prepared in a 96-well plate, and coated nanoparticles were incubated for 48 h in Eppendorf tubes and then transferred to the 96-well plate. The nanoparticles were not purified before the experiment. Next, 30% hydrogen peroxide was added to the wells (final concentration of 3%) and absorbance was measured at 800 nm every 3 min for 2 h using a Synergy H1 microplate reader (BioTek). Results were normalized to the initial value at *t*_0_.

### Characterization of Nanoparticles

Size and Zeta Potential: Solutions were diluted 1:10 in DDW and measured using a Zetasizer Nano ZS instrument (Malvern Panalytical, Malvern, UK). The results are reported as the average of 3 independent measurements ± the deviation from the mean. The uniformity of the size distribution was recorded as the polydispersity index (PDI) obtained with the particles’ size.

Drug Loading: Nanoparticles diluted 1:10 in acetonitrile solution were measured with ultraperformance liquid chromatography (Acquity arc UHPLC, Waters Corp., Milford, MA, USA) using a CORTECS C18 (4.6 × 50 mm 2.7 μm) column.

Cryogenic Transmission Electron Microscopy (Cryo-TEM): Samples were prepared with the kind assistance of The Technion Center for Electron Microscopy of Soft Matter (Department of Chemical Engineering, Technion). A small drop (3 μL) was applied to a perforated carbon film supported on a standard TEM grid (Ted Pella). The drop is then blotted into a thin liquid film spanning the holes by bringing it into contact with a piece of filter paper. All steps were carried out in the Controlled Environment Vitrification System (CEVS) chamber at ambient temperature, saturated with water vapors.^26^ The specimen was plunged into freezing ethane at −183 °C and loaded into a TEM cooling holder (“cryo-stage”) using a dedicated “transfer station”. During imaging, the holder tip is kept at approximately −180 °C to avoid sublimation and to preserve the supercooled state of the vitreous ice. The imaging was performed with the minimum electron dose possible (about 10 e^–^/Å^2^) on a Talos F200X FEG TEM (Thermo Fisher) using a “Volta phase plate” for contrast enhancement.^27^ The micrographs were recorded with a direct Falcon electron camera using TIA software.

High-Resolution Scanning Electron Microscopy (HR-SEM): Five microliters of the nanoparticle solution were applied to a silicon wafer and placed in a desiccator under vacuum for 72 h for dehydration. HR-SEM imaging was performed by the Technion EMC, the Electron Microscopy Center (Department of Materials Science and Engineering, Technion), on a Zeiss Ultra Plus high-resolution SEM (Oberkochen, Germany), equipped with a Schottky field-emission gun. Specimens were imaged at an acceleration voltage of 1.3 kV and a working distance of 3 mm. The Everhart Thornley (“SE2”) secondary electron imaging detector was used.

Energy Dispersive Spectroscopy (EDS): Samples were prepared the same as for the HR-SEM samples. Analysis was performed using a Zeiss Ultra-Plus FEG-SEM equipped with an EDS detector (silicon drift detector: 80 mm^2^ active area, 127 eV energy resolution, X-MAX, Oxford). The instrument was operated at an accelerating voltage of 4 kV and a working distance of 3.3 mm. Elemental spectra and mapping were acquired from representative regions of each sample. Data were collected and processed using the instrument’s dedicated software. All spectra were compared against library standards for the qualitative and quantitative determination of elemental composition.

Imaging Fluorescent Nanoparticles and Drug Aggregates: The wells were imaged in the DAPI channel (Ex. 377 nm, Em. 447 nm) of a LionHeart (BioTek) with 10 ms exposure, 10% digital gain, 100% LED intensity, and image-based autofocus as well as in the bright field channel. To quantify the AIE effect, an analysis protocol of image statistics – total intensity was used from the supplier of the image analysis software, Gen5+ Data Analysis. To quantify the number of aggregates, we used ImageJ’s “analyze particles” function on the bright field channel.^28^

### Drug Release Profile

Trametinib nanoparticles were incubated in serum-containing Dulbecco’s Modified Eagle Medium (DMEM, pH 7.4) at 37 °C at a concentration equivalent to 0.45 mg/mL of drug. The amount of released drug was determined by centrifugation at 14000 rcf for 10 min in AmiconUltra (Sigma-Aldrich) centrifugal filters (0.5 mL, 100 KD). The drug was extracted from the supernatant into an acetonitrile solution and measured in UHPLC.

### Decision Tree

The chemical structures of the screened drugs were retrieved from the ChemSpider web server as mol files. All available molecular descriptors in Dragon software (version 7, Talete SRL, Milan, Italy, 2007) were calculated for the input drugs in order to find the best possible correlation. The training data set included 30 drugs, and the test data set included 10 drugs. Principal component analysis was performed with the principle component analysis (PCA) built-in function in Dragon for the training and validation data sets separately.

### Cell Cultures

HCT116 cells were a kind gift provided by the lab of Moshe Elkabets (Ben-Gurion University, Israel), and the K7M2 cell line was kindly donated by the Yuval Shaked laboratory (Technion, Israel). Both cells were cultured in DMEM (Sartorius, Goettingen, Germany) supplemented with 10% fetal bovine serum (FBS), 2 mM l-glutamine, penicillin G sodium salt (100 units/mL), and streptomycin sulfate (0.1 mg/mL) (pen-strep). Cells were incubated at 37 °C with 5% CO_2_ and 65% humidity.

Cell Viability Assay in 2D: Both cell lines were seeded in 96-well plates at a 30% confluency (5000 cells per well) and allowed to adhere for 24 h. All drugs were dissolved in DMSO, and nanoparticles were suspended in DDW and added to the wells. Untreated cells (control) were used to establish the 100% viability. The effect on cell viability was measured with the Promega (Madison, WI, USA) CellTiter-Glo (CTG) 2D cell viability assay.

Cell Viability Assay in 3D: HCT116 and K7M2 were seeded in 96-well round-bottom ultralow attachment plates (1000 and 700 cells per well, respectively) and allowed to form spheroids for 72 h. All drugs were dissolved in DMSO; nanoparticles were suspended in DDW and added to the wells. Untreated cells (control) were used to establish 100% viability. The effect on cell viability was measured with a Promega CellTiter-Glo (CTG) 3D cell viability assay.

Nanoparticle Penetration: K7M2 spheroids were formed and transferred to a glass-bottom black-sided flat 96-well plate for 24 h to allow attachment. Nanoparticles were added to the wells (0.1 mg/mL) and incubated for 90 min, then washed with 100 μL of X2 PBS and left in Hanks’ Balanced Salt Solution (HBSS). 50 μL of Hoechst (final concentration 4.8 × 10^–3^ mg/mL) in DMEM was added, and after 15 min of incubation the spheroids were imaged in the LionHeart using a DAPI filter (Ex. 377 nm, Em. 447 nm) and Cy7 filter (Ex. 716 nm, Em. 809 nm) with 10 ms exposure, 10% digital gain, 100% LED intensity, and image-based autofocus. To quantify the intensity levels, an analysis protocol of image statistics – total intensity was used from the supplier image analysis software Gen5+ Data Analysis.

### Animal Studies

All of the animal studies were conducted according to protocols approved by the Institutional Animal Research Ethical Committee at the Technion – Israel Institute of Technology. Mice were maintained and treated in accordance with the Institutional Animal Research Ethical Committee at the Technion – Israel Institute of Technology.

Colorectal Carcinoma Xenograft Studies (HCT116): Six-week-old female Hsd:Athymic Nude-Foxn1nu mice purchased from Envigo RMS were injected with 5 × 10^5^ HCT116 human colorectal carcinoma cells subcutaneously in 100 μL of culture media/Matrigel (Corning, NY, USA) at a 1:1 ratio. Animals were randomized into groups, with *n* = 8–10 tumors per group. Animals were treated intraperitoneally (I.P.) with trametinib-IR783 NPs or trametinib-IR783 + DA coating NPs (20 mg/kg) or trametinib in DMSO (2 mg/kg) twice a week. Tumor size was measured with a digital caliper, and tumor volumes were calculated using the following formula: (length × width^2^) × (π/6). Animals were euthanized by using CO_2_ inhalation. Mice were housed in air-filtered laminar flow cabinets with a 12 h light/dark cycle and food and water *ad libitum*.

### Statistical Analysis

Statistical analysis for this work was performed using GraphPad Prism (GraphPad 9 Software). A Welch’s unpaired *t*-test was conducted to compare different groups. The significance level was set at *p* < 0.05. Independent experiments were conducted with a minimum of three replicates to allow for statistical comparison. Error bars represent the standard error of the mean (s.e.m.), and *p* values are indicated in the figure captions and main text. The survival plot was analyzed using the Mantel–Cox log-rank test. For all *in vivo* experiments, the sample size was at least *N* = 6 tumors per treatment group. These sample sizes were chosen based on previous literature and our own expertise. Animal cohorts were randomly selected. Investigators were not blinded.

## Results and Discussion

### Coating Protocol and Drug Screen

To achieve a simple and efficient coating of IR783-stabilized drug nanoparticles, we leveraged the fact that nanoprecipitation occurs in alkaline bicarbonate buffer, which provides optimal conditions for dopamine polymerization. This strategic approach ensures sequential nanoparticle formation, followed by immediate coating initiation within a single reaction vessel, streamlining the synthesis process. The alkaline environment (pH 8.5) first promotes drug–dye coassembly into stable nanoparticles, then facilitates dopamine oxidation and subsequent PDA coating formation over 48 h at room temperature. PDA’s adhesive properties, based on hydrophobic interactions, support the nanoparticles structure for extended stability.^18^ Following the coating process, a single centrifugation step effectively removes DMSO, unreacted dopamine monomers, and unencapsulated drug and dye, yielding purified PDA-coated nanoparticles in a straightforward one-pot synthesis.

We optimized our process in the known pH level of 8.5 in bicarbonate buffer as most reported protocols for dopamine polymerization use Tris buffer.^10^ To validate the necessity of each component in our system, we systematically tested various combinations using sorafenib as a model drug (Figure S1A and B). We investigated what occurs when dopamine polymerization proceeds in the presence of dye alone (without drug nanoprecipitation) as well as drug nanoprecipitation without dye (regular precipitation conditions). These control experiments revealed that only the complete three-component system—drug, IR783 dye, and PDA coating—produced stable nanoparticles, demonstrating the essential role of each element in achieving successful particle formation and coating.

To optimize coating conditions, we leveraged the characteristic brown coloration and 490 nm absorbance (unique to PDA in this system) to monitor polymerization kinetics (Figure S1C–E). Time-course studies revealed that while a 24 h coating produced higher absorbance values, suggesting increased PDA content, these formulations exhibited poor colloidal stability over extended storage. They increased in size over the first 3 days and eventually aggregated and precipitated. This finding indicated that longer polymerization times are necessary to achieve both an adequate coating and stable nanoparticle dispersions. As sorafenib is known to be very stable in these dye nanoparticles without coating,^5^ we concluded that lower stability in this model drug indicates that 24 h is a less favorable polymerization time.

The polymerization depends on the presence of oxygen;^8^ however, we observed that oxygen interrupts the formation of stable nanoprecipitated nanoparticles. Therefore, we first looked for optimal conditions for the coating process. We tested this process with three drugs that form stable nanoparticles with IR783 (sorafenib, paclitaxel, and vismodegib) and one that forms unstable nanoparticles (trametinib).^5^

Since polydopamine does not have a specific absorbance peak but a broad spectrum,^10^ we continued to focus on the absorbance at 490 nm to assess the coating quality since it does not interfere with the absorbance of IR783 in near-infrared or UV absorbance of aromatic drugs and was significant in all the experiments (Figure S2). Shaking affected the process; for most formulations, the signal at 490 nm was stronger without shaking, indicating improved coating (Figures 1A, S3). Also, the stability of most nanoparticles was harmed by the shaking process (Figure 1B and C). Therefore, we decided to continue the experiments without shaking. Already in this small three-drug set experiment we noticed a differential coating yield, with higher coating for paclitaxel and trametinib and almost no coating for vismodegib, and we expended it to a 30-drug screen in order to build a prediction model (Figure 1D and E). In Figure 1D a visible change in the nanoparticle color is shown, while Figure 1E shows a change in the absorbance at 490 nm following the coating process compared to uncoated IR783 nanoparticles. Dark brown nanoparticles were considered densely coated, while nanoparticles that did not change their color after the process were considered not coated. 17AAG was one of the drugs that did not seem to coat, and it has a negative difference, whereas poantinib, nilotinib, and osimertinib had a strong coating and high positive value. However, celecoxib was not coated yet had a high positive value and bicalutamide had weak coating and a negative difference. A possible explanation for celecoxib’s high absorbance value after the coating process might be poor stability and aggregation of the nanoparticles.

**Figure 1:**
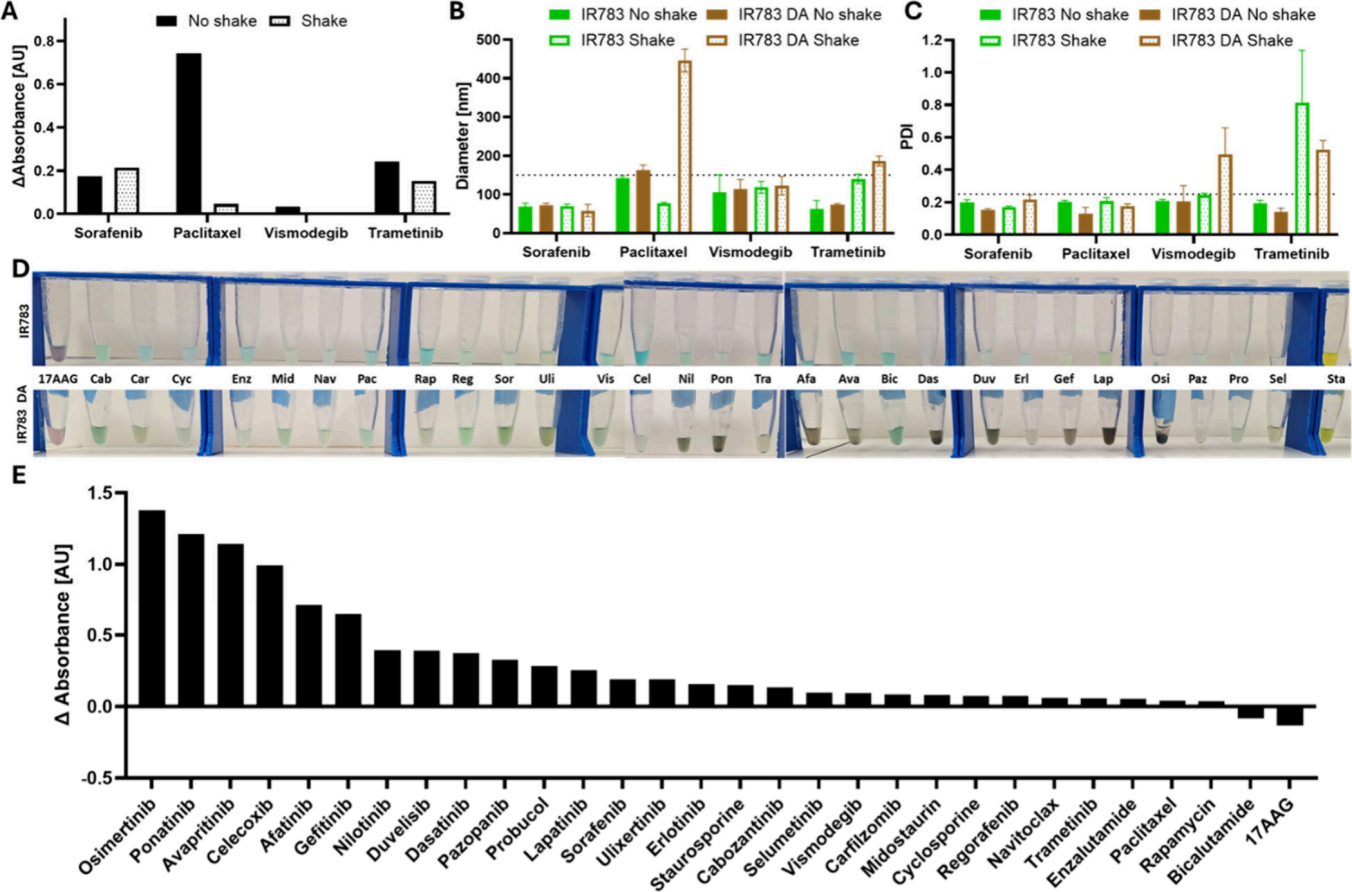
Protocol verification and drug screen. (A) difference in the absorbance measured at 490 nm between shaken and nonshaken nanoparticles. (B, C) Size (B) and PDI (C) of sorafenib, paclitaxel, vismodegib, and trametinib nanoparticles. (D) Images of the nanoparticles with (bottom) and without (top) polydopamine coating. (E) Difference in the absorbance at 490 nm for coated nanoparticles compared to noncoated.

We next sought to both understand and predict the differential coating phenomenon observed with different drug structures by using cheminformatics approaches. Based on both the difference in absorbance at 490 nm and visible color changes, we defined a binary classification variable (VAR6) as input for molecular descriptors, Dragon software (Figure 2A). The software calculated all 4885 available molecular descriptors (as opposed to python RDKit 208 descriptors) and determined their correlation with our experimental coating variable. The chemical descriptors exhibiting the highest correlation with coating efficiency were F02­[N–N] (frequency of N–N at a topological distance 2), SdsN (sum of dssN E-states for nitrogen atoms), NdsN (number of dssN atoms), and N% (percentage of nitrogen atoms).

**Figure 2:**
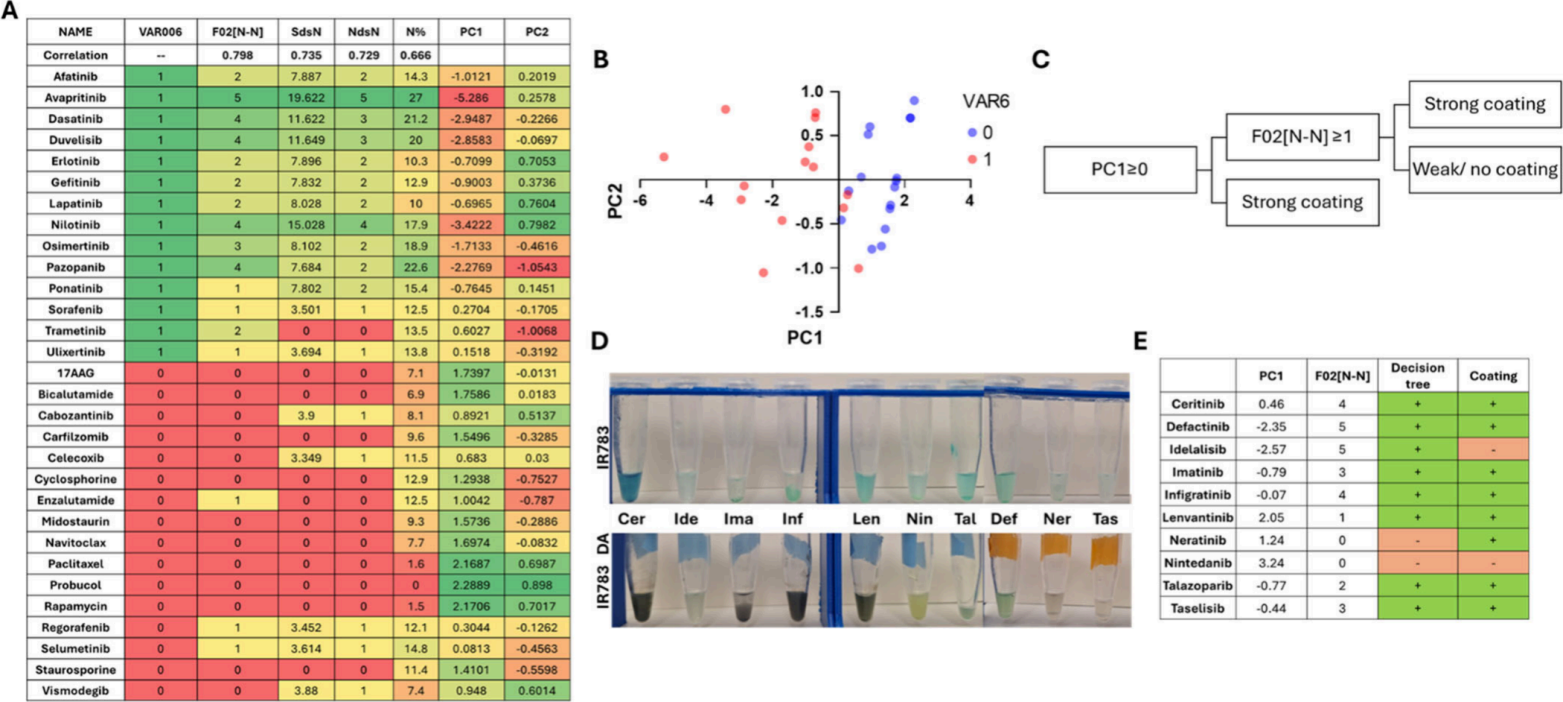
Building and verifying the model. (A) The parameters used for building the PCA and prediction model. (B) Principal component 1 and 2 scores for each drug; the corresponding VAR6 value is indicated in blue/red. (C) The decision tree hierarchy. (D) Images of the validation nanoparticles with (bottom) and without (top) polydopamine coating. (E) Parameters for the decision tree validation, prediction, and coating scores.

In order to construct a decision tree, we preformed principle component analysis (PCA) on those descriptors.^29,30^ As discussed in the work of Todeschini et al.,^31,32^ the *k* correlation index can assist in selecting the number of significant principal components (PCs), based on the formula

KL=int⁡1+(p−1)(1−k)

where *k* = the *k* correlation index, int = the nearest integer upper value to *k*, *p* = the number of variables, and KL = maximum number of theoretical significant principal components.

In our PCA the *k* correlation index was 0.862, meaning we should consider only PC1 as a significant PC. This can also be seen by the eigenvalues and proportion of variance; PC1 can explain 89.62% of the variance (Figure S4). We based our decision tree on PC1 and the topological distance between nitrogens in the molecule (F02­[N–N]) which had the highest correlation with VAR6 (Figure 2B and C). Later, the validation set underwent the same chemical descriptor calculations and PCA. The decision tree correctly predicted the coating level for 80% of the drugs in this set (Figure 2D and E).

Based on this decision tree, we understood that the coating is dependent not only on nonspecific hydrophobic interactions or π–π stacking but also on the presence of nitrogens in the molecule and their chemical bonds. One good example for this is the drug probucol, a highly hydrophobic drug without nitrogen atoms. Its coating was very weak and the difference in the absorbance was relatively low (0.238, Figure 1E). All strongly coated drugs had N% > 10 and at least once the motif “N–C–N”.

### Nanoparticle Characterization

We then continued to thoroughly characterize the coating and coated nanoparticles. We first sought to understand whether the coating is incorporated with the IR783 dye or covering it. In Figures 3 and S5 we show the decay of IR783’s absorbance due to a reaction with hydrogen peroxide (H_2_O_2_).^33,34^ The profile for each drug is dependent of the drugs’ structure; for example, cyclic drugs (e.g., 17AAG) form more porous nanoparticles, resulting in faster decay of the absorbance signal.^5^ Our assumption is that a strong and uniform coating will limit the penetration of the small H_2_O_2_ molecule to the nanoparticles, therefore delaying or preventing the reaction. As expected, nanoparticles that had stronger coating, like ponatinib, were more protected than nanoparticles with weaker coatings like cabozantinib, even though both are not cyclic. We hypothesized that there might be a reaction between polydopamine and IR783 and formation of drug-free nanoparticles because even in the vehicle-only wells, we noticed a certain level of protection over the dye.

**Figure 3:**
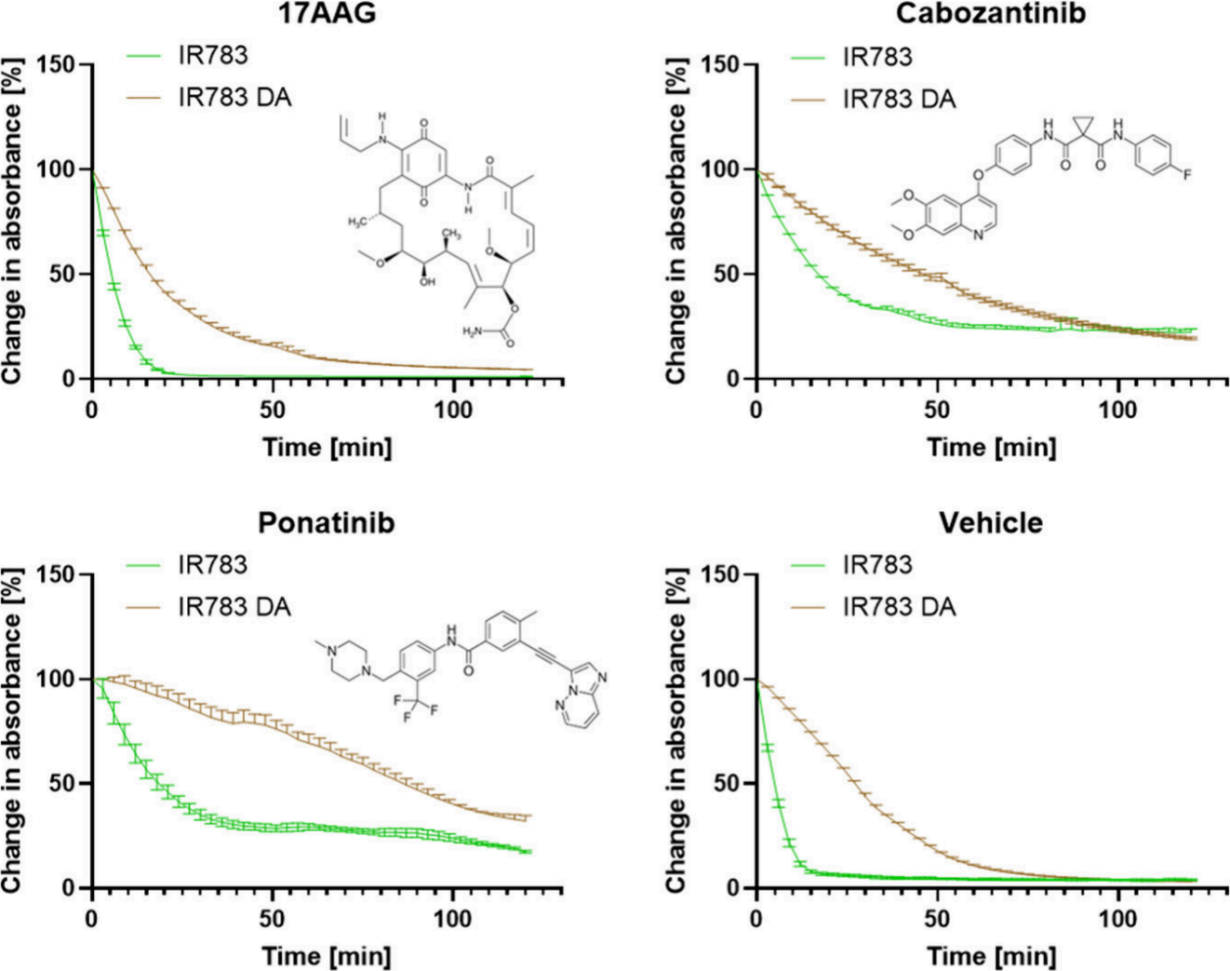
Change in absorbance at 800 nm over 2 h in H_2_O_2_ 3% solution for coated and noncoated nanoparticles.

To further characterize the differential coating phenomenon, we focused on two drugs that benefitted from the coating: trametinib and dasatinib. We compared coated and noncoated nanoparticles for each of the drugs and investigated them using different methods.

First, we examined trametinib nanoparticles, chosen for their improved stability through coating. Trametinib is an orally administered MEK inhibitor approved by the FDA for melanoma and colorectal cancer treatment. However, in many cases resistance to the drug is developed over time, highlighting the need for a more effective treatment.^35^

In Figures 4A and S6 we showed representative cryo-TEM images of coated and noncoated nanoparticles. We noticed a weaker contrast in the coated nanoparticles, but their overall size and morphology were similar. When we looked at the nanoparticles’ size over time, we saw that the coated nanoparticles are stable for 3 days and are more uniform in population. The sizes measured by DLS on the first day correlate well with the cryo-TEM images (Figures 4B,C and S7). Quantitative analysis of TEM images yielded average sizes of 94 nm for noncoated and 138 nm for coated nanoparticles. We hypothesize that the improved stability is because PDA functions as a molecular adhesive, stabilizing the nanoaggregate through mostly π–π stacking interactions with both aromatic drug molecules and IR783, along with hydrogen bonding and hydrophobic forces. This “molecular glue” effect prevents premature particle dissociation and aggregation, as evidenced by the enhanced stability and washing resistance of coated nanoparticles.

**Figure 4:**
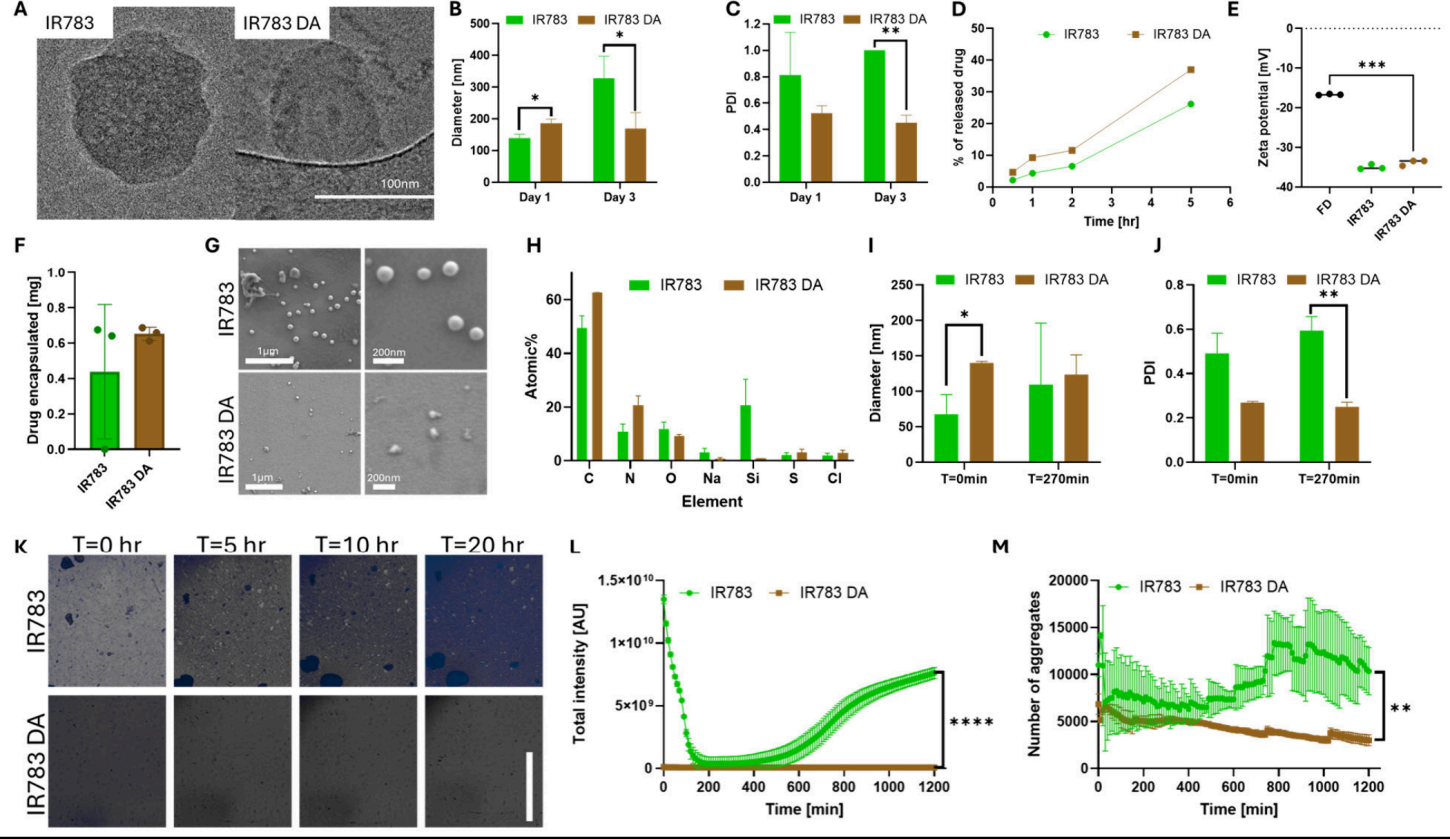
Characterization of trametinib and dasatinib nanoparticles. (A) CryoTEM images of noncoated (top) and coated (bottom) nanoparticles, scale bar = 100 nm. (B, C) Size (B) and PDI (C) of the nanoparticles over time, *p* < 0.04. (D) Kinetics of drug release from the nanoparticles. (E) Zeta potential of the free drug aggregates and the nanoparticles, *p* = 0.0003. (F) Measured weight of dasatinib encapsulated in nanoparticles across different batches. (G) SEM images of noncoated (top) and coated (bottom) nanoparticles. (H) EDS results for the atomic % of each element in the SEM samples. (I, J) Change in the nanoparticles’ diameter (I) and PDI (J) over 270 min, *p* < 0.04. (K) Representative images of the aggregation process over 20 h in noncoated (top) and coated (bottom) nanoparticles, scale bar = 1000 μm. (L) Intensity of the blue signal (377, 447) over the aggregation process, *p* < 0.0001. (M) Averaged number of aggregates counted over 20 h, *p* = 0.0079.

To verify that the coating does not harm the nanoparticles’ ability to release the drug, we conducted a 5 h release kinetics experiment (Figure 4D). The drug release profile from the coated nanoparticles followed a similar trend to that of the noncoated nanoparticles. Zeta potential can be used as an indicator for stability, and values ranging from ±30 to ±40 mV are considered moderately stable.^36^ In Figure 4E, we show the zeta potential measurements of the free drug colloids compared to the two types of nanoparticles. The measurements did not indicate a significant difference in the surface zeta potential between the two types. Statistical analysis (unpaired *t* test) confirmed no significant difference between coated and noncoated samples (*p* > 0.1), but their charge is significantly lower than that of free drug aggregates and correlates with the expected values of moderately stable nanoparticles.

The next drug studied was dasatinib, a BCR-ABL inhibitor approved for treating chronic myeloid leukemia and Philadelphia-chromosome-positive acute lymphoblastic leukemia. It is mostly used as a second line therapy for patients resistant to imatinib.^37^

Coated dasatinib nanoparticles were more uniform in size and stable for a longer period. Moreover, the drug encapsulation ratio was constant between different batches (Figure 4F). HR-SEM scans (Figures 4G and S8) revealed changes in nanoparticle morphology, with coated nanoparticles appearing less spherical and more heterogeneous in their morphology, suggesting that polydopamine successfully coated the nanoparticles as hypothesized and altered their surface characteristics. In Figure 4H, we showed that EDS results for these nanoparticles revealed no significant change in the elemental composition of the nanoparticle. However, we observed an increase in carbon and nitrogen’s relative percentage, both of which are major components of polydopamine. An increase in the relative percentages of these two elements was reported in other works and is dependent on the coated surface.^38^ We observed an increase of approximately 10% for nitrogen and 13% for carbon. Even though the naïve nanoparticles are smaller in size, their population is much more dispersed, and they began to significantly aggregate after only 5 h (Figure 4I,J). The HR-SEM scans also correlated to the DLS measurements; quantitative analysis of the images shows an average size of 95 nm for the coated nanoparticles and 88 nm for the noncoated nanoparticles (Figure S7).

The final step for dasatinib nanoparticles was to characterize their aggregation profile (Figure 4K–M). This analysis was based on aggregation-induced emission (AIE),^39,40^ an increased fluorescence emission in the aggregated state compared to the soluble state. In its aggregated state, dasatinib is fluorescent under a DAPI filter (Ex. 377 nm, Em. 447 nm). We imaged for 20 h and saw that the coated nanoparticles were not only smaller overall but also more uniform in size distribution, consistent with the DLS measurements. We monitored the AIE effect of dasatinib as an indicator of significant aggregation process and received a significantly higher signal in the noncoated wells, which increased over the time of the experiment. The initial decrease at the beginning of the experiment can be explained by the time it takes particles to sink and by the time it takes the wells to stabilize their signal.

### *In Vitro* Cell Culture Experiments

We tested the formulations on two types of solid tumor cells: K7M2, an osteosarcoma cell line, and HCT116, a colon cancer cell line. Osteosarcoma is difficult to treat because of the high variability in the driving mutations between patients. K7M2 is a murine cell line; it does not have a well-defined driving mutation similar to other osteosarcoma cell lines. In a functional screen, it showed sensitivity to different kinase inhibitors, many of which had good PDA coating. We compared dasatinib, imatinib, and sorafenib and chose dasatinib as a model drug since it was significantly more potent in 3D spheroids (Figure S9). As mentioned, dasatinib is a BCR-ABL inhbitor, but it has several off targets such as SRC, which is upstream of the Hippo pathway, which is speculated to be driving tumor progression in osteosarcoma.^41,42^

As shown in Figure 5A, in 2D cell cultures, the nanoparticle-encapsulated drug exhibited significant improved efficacy compared to the free drug (FD). This advantage was not observed in 3D spheroids (Figure 5B), due to nanoparticles’ limited penetration compared to a small molecule.^43^ We noticed that even though there is no definite change in the overall efficacy, the mechanism of cell death differs with the treatment (Figure 5C). We hypothesized that the cells go through necrotic cell death in the presence of the free drug, apoptotic cell death in the presence of IR783 nanoparticles, and a type of autophagy in the presence of the coated nanoparticles.^44^

**Figure 5:**
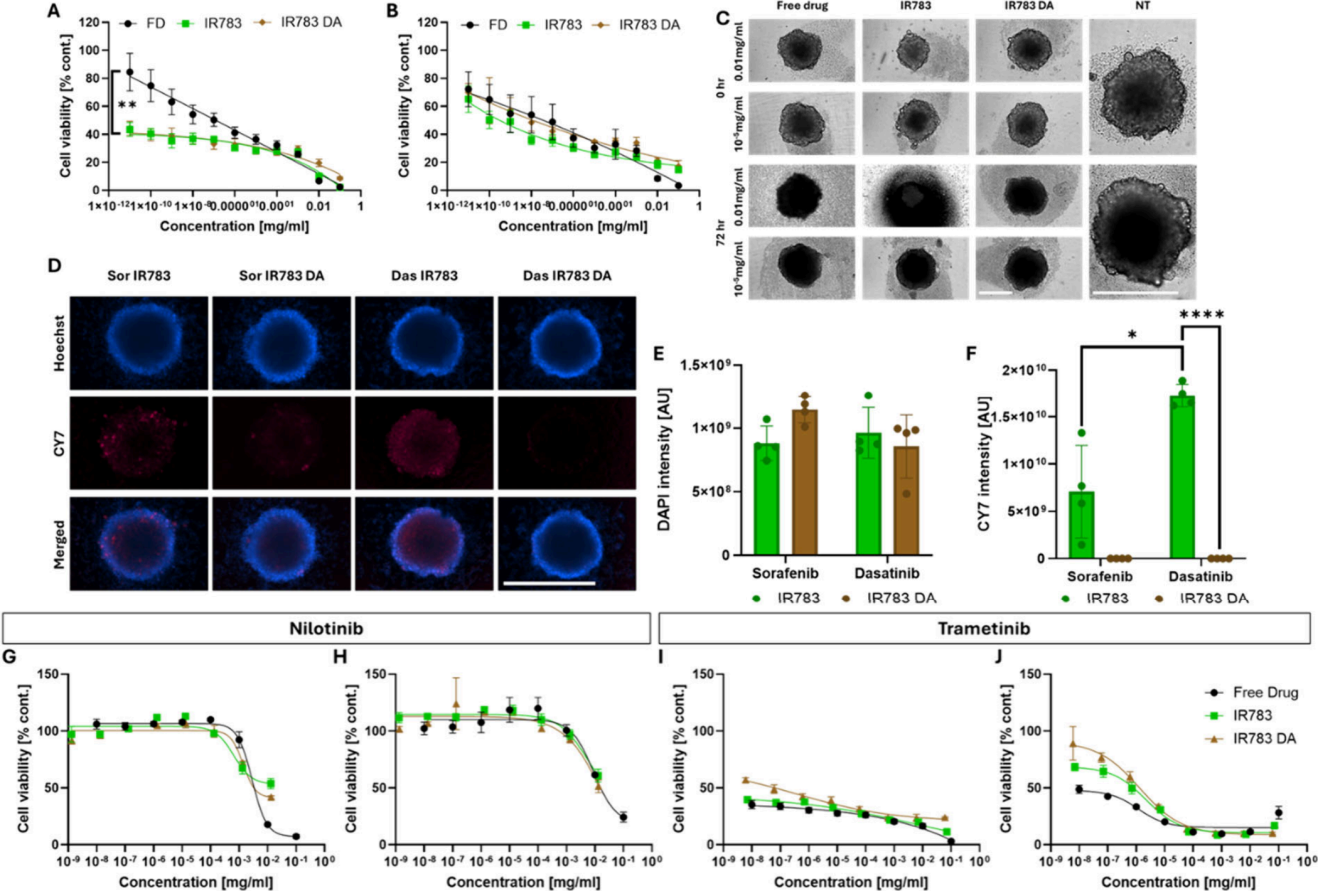
*In vitro* assays on K7M2 and HCT116 cell lines. (A, B) The efficacy of dasatinib as free drug on K7M2 2D (A) and 3D (B) cell cultures, compared to coated and noncoated nanoparticles, *p* = 0.0012. (C) K7M2 spheroid images before and after treatment compared to nontreated control. Scale bars = 400 nm. (D) Imaging nanoparticles’ penetration of K7M2 spheroids, blue = Hoechst (377, 447), pink = IR783 (716, 809), sor = sorafenib, das = dasatinib, scale bar = 500 μm. (E) Quantitative analysis of DAPI intensity in K7M2 spheroids. (F) Quantitative analysis of CY7 intensity in K7M2 spheroids, *p* < 0.02. (G–J) Viability results of HCT116 cells with nilotinib (G, H) and trametinib (I, J). 2D results are shown on the right and 3D results are shown on the left for each drug.

Based on IR783 fluorescence in the CY7 filter (Ex. 716 nm, Em. 809 nm),^45^ we analyzed the penetration depth of the nanoparticles. Figures 5D–F present a comparison between two drugs, dasatinib and sorafenib. We chose sorafenib as a comparison since it coats well and it forms stable nanoparticles with and without PDA coating. The spheroids were incubated with the nanoparticles for 90 min, after which Hoechst solution was added to verify the spheroids’ overall viability, and then the spheroids were imaged. Even though dasastinib has an AIE effect and its nanoparticles are fluorescent in the DAPI filter, the intensity is much weaker compared to the Hoechst solution, and no significant difference in the DAPI intensity was noted (Figure 5E). When observing the CY7 filter, there is a very significant difference between coated and noncoated dasatinib nanoparticles, hinting at a quenching effect or masking of the IR783 by the coating. We did not notice a significant difference in sorafenib nanoparticles, probably due to a large standard deviation in the noncoated nanoparticles. The higher signal observed in dasatinib nanoparticles is likely due to their greater incorporation of IR783 dye, as drug-dependent differences in dye loading are known to occur^23^ (Figure 5F).

For the *in vivo* studies in the well-established model of HCT116,^46–48^ we tested four drugs. Since the driving mutation in HCT116 is a KRAS mutation,^49^ trametinib and nilotinib, which benefit from the coating, were relevant and expected to be potent on this cell line. Trametinib is a MEK inhibitor that is downstream to KRAS,^35,50^ and nilotinib inhibits BCR-ABL, which is upstream to KRAS.^51^ We also tested two more drugs, ulixertinib and midostaurin. Midostaurin was not coated well, while ulixertinib was well coated, but there was no clear advantage to the coated nanoparticles over the IR783 nanoparticles. Both were less potent than trametinib, and therefore we did not pursue them any further (Figure S10). As seen in Figure 5G–J, trametinib had superior efficacy in both 2D and 3D cell cultures, and we decided to explore this drug further *in vivo* and look for more interesting advantages.

### *In Vivo* Experiments

Based on the promising *in vitro* results with trametinib, we proceeded to *in vivo* studies, aiming to assess whether dopamine coating could improve biodistribution and therapeutic efficacy while reducing toxicity. We hypothesized that since PDA has excellent cell adhesion properties it could slow the spread of the nanoparticles and will result in a more controlled release profile.^10,52^ The change in the pharmacokinetics could result in fewer systemic side effects and higher effective doses in the tumor.^53^

Mice were treated with trametinib free drug at 2 mg/kg, a standard dose commonly used in murine models.^54^ In contrast, both coated and noncoated nanoparticles enabled administration of a substantially higher dose (20 mg/kg), owing to their ability to mitigate side effects and increase the maximum tolerated dose.^55^ The main advantage of the PDA coating seen in this experiment is extended shelf life formulation stability, which improved dramatically and enabled treatment at several days from preparation, while the uncoated had to be injected within 2 h of preparation.

Biodistribution profiles 24 h after the injections were comparable (Figure 6A–C); there is some increase in accumulation of the coated nanoparticles in the liver, but it is not significant (*p* > 0.05). The results shown are normalized to the tumor intensity due to the masking of the coating, as was discussed in the *in vitro* section. Furthermore, coated nanoparticles demonstrated efficacy, survival outcomes, and safety similar to those of noncoated IR783 nanoparticles (Figures 6D–F and S11). However, the key advantage of the PDA-coated nanoparticles lies in their superior stability, which allows for advance preparation and facilitates administration.

**Figure 6:**
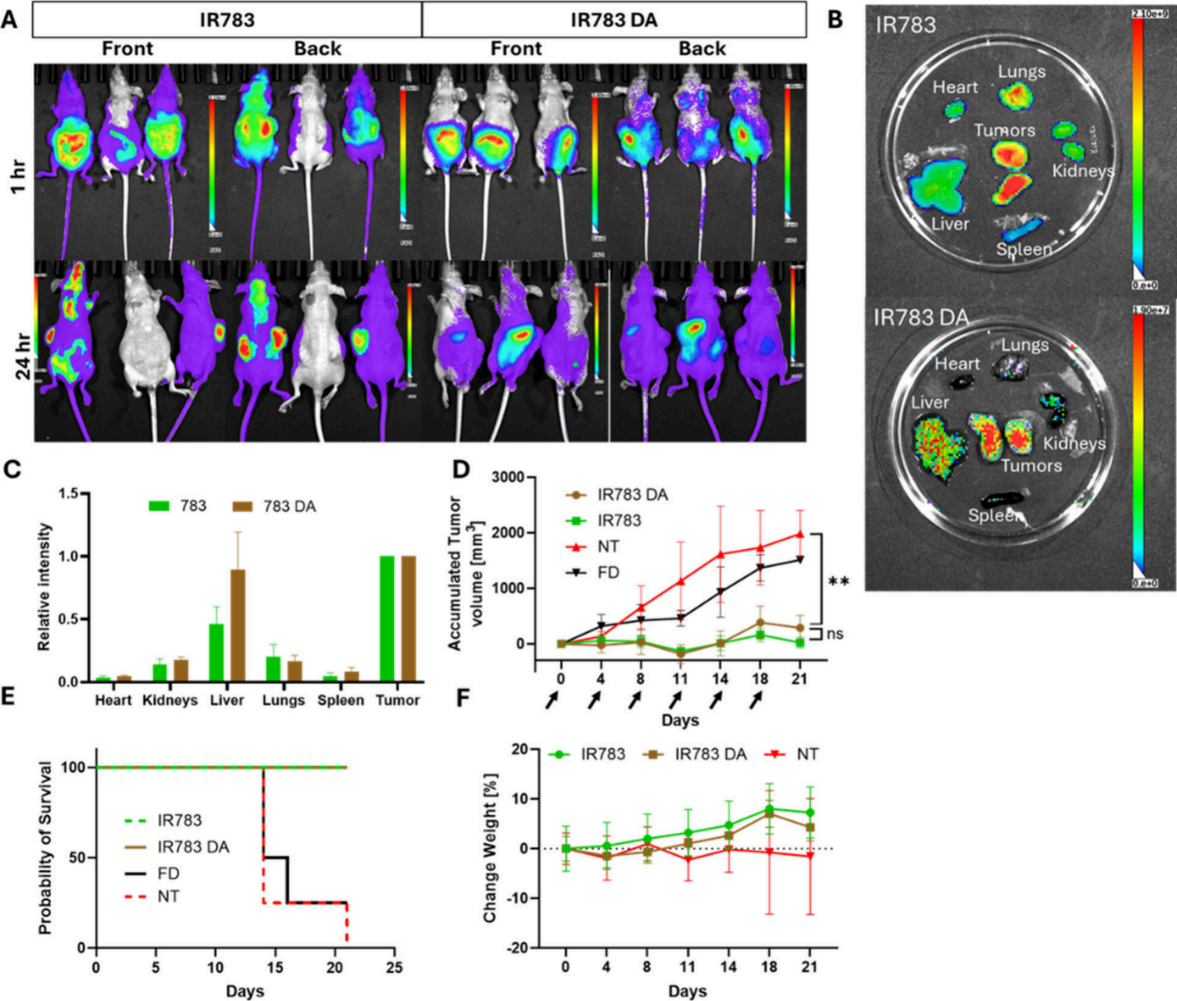
*In* *vivo* studies in HCT116 xenografts. (A) IVIS images of treated mice 1 h (top) and 24 h (bottom) after trametinib nanoparticle injection. (B) IVIS images of organs 24 h after nanoparticle injection. (C) Fluorescence intensity of the single organs normalized to the tumor intensity (*n* = 3). (D) Change in accumulated tumor volume over time (*n* = 4), *p* < 0.0012. (E) Kaplan–Meier curves of treated and nontreated mice (*n* = 4). (F) Change in mice weight, normalized to day 0. NT = nontreated, FD = free drug, IR783 = IR783 stabilized trametinib nanoparticle, IR783 DA = dopamine coated IR783 stabilized trametinib nanoparticle.

The delayed release of the coated nanoparticles from the injection site could be exploited in the future for photothermal therapy. In this treatment, a near-infrared wavelength is used on photosensitizing agents to turn light into heat that can damage nearby tissues. PDA has strong absorption in the relevant region of 700–1300 nm, making the coated nanoparticles good candidates for such treatment, as was shown in several works that include PDA.^56,57^ While both coated and noncoated nanoparticles demonstrated superior *in vivo* efficacy over free drug formulation, the PDA coating provides additional advantages through its versatile functionalization platform. The structure-selective coating behavior enables the prediction of coating efficiency from molecular descriptors, transforming PDA stabilization from an empirical to a rationally designable approach. Additionally, the reactive catechol/quinone chemistry of PDA enables straightforward conjugation of targeting ligands (such as folate or RGD peptides), imaging agents, or other functional molecules through Michael addition or Schiff base formation under mild conditions. Thus, PDA coating addresses current formulation challenges of stability and handling while providing a predictable, functionalizable platform for next-generation targeted delivery systems. In addition, the PDA coating is economical and practical for pharmaceutical scale-up. Dopamine hydrochloride is inexpensive ($50–100 per gram, milligrams per batch), and the process requires only dopamine and bicarbonate buffer, with no expensive equipment or organic solvents needed. This green chemistry approach uses biocompatible materials with established safety profiles and scales easily from the laboratory to manufacturing, making it attractive for commercial production.

All the animal studies were conducted according to protocols approved by the Institutional Animal Research Ethical Committee at the Technion – Israel Institute of Technology, ethics number IL0740620. Mice were maintained and treated in accordance with the Institutional Animal Research Ethical Committee at the Technion – Israel Institute of Technology.

## Conclusions

Our work demonstrates, for the first time, that PDA exhibits structure-selective coating behavior on drug nanoparticles, a finding that distinguishes it from the universal coating assumption in previous literature. This selectivity enables a predictive formulation design using molecular descriptors and rational selection of drug candidates for PDA stabilization. Compared to alternative stabilization approaches such as polysaccharide integration (fucoidan, dextran), lipid coatings, or polymer conjugation (PEGylation), our PDA approach offers a simple, aqueous-based, biocompatible process with predictable behavior. Using a combination of systematic screening and molecular descriptors, we constructed a predictive decision tree that accurately classified the coating potential in 80% of validation compounds, primarily guided by nitrogen content and bonding topologies.

Comprehensive characterization revealed that PDA coating enhanced nanoparticle stability, reduced aggregation, and preserved drug release kinetics without compromising the loading efficiency. Model drugs such as trametinib and dasatinib exhibited improved colloidal properties and surface morphology following PDA coating. We hypothesized that there is some physical interaction between the stabilizer, IR783, and the PDA coating which led to quenching when imaged in fluorescent microscopy both *in vitro* and *in vivo*.

*In vivo* experiments using trametinib nanoparticles in HCT116 xenografts demonstrated that the PDA-coated nanoparticles were as good as noncoated nanoparticles for tumor growth inhibition and survival outcomes.

Together, these findings establish that PDA coating is inherently specific and chemically dependent, and rational prediction of coating efficiency can guide the development of more effective and stable nanoparticle-based drug delivery systems, which can then be easily conjugated with nucleophiles containing ligands.

## Supplementary Material



## Notes

Selecting the conditions, UV–vis spectra, histograms and images of formulations, PCA% EV and loadings, additional drugs’ IR783’s absorbance decay, cryo-TEM image, histograms for electron microscopy, HR-SEM images, kinase efficacy on K7M2, drug efficacy on HCT116, individual tumor volumes (PDF)

## Abbreviations

AIE: aggregation-induced emission
CEVS: controlled environment vitrification system
CTG: CellTiter-Glo
DDW: double-distilled water
DMEM: Dulbecco’s modified Eagle medium
DMSO: dimethyl sulfoxide
EDS: energy dispersive spectroscopy
FBS: fetal bovine serum
HBSS: Hank’s balanced salt solution
HR-SEM: high-resolution scanning electron microscopy
I.P.: intraperitoneal
PCA: principle component analysis
PDA: polydopamine
PDI: polydispersity index
TEM: transmission electron microscopy
UHPLC: ultraperformance liquid chromatography
