# Supplementary material for: Structure-Selective Polydopamine Coating on Drug Nanoparticles
Source: ACS Appl Mater Interfaces. 2026 Jan 26;18(4):6452–63. doi: 10.1021/acsami.5c19920 (PMC12884477; doi:10.1021/acsami.5c19920)
Supplement: Supplementary file 1 [file am5c19920_si_001.pdf]

Supporting Information

Structure-Selective Polydopamine Coating on Drug Nanoparticles

Danna Niezni[1], Dana Meron-Azagury[1], Maytal Avrashami[1], Orr Bar-Natan[1,2], Yosi Shamay[1]*

[1]Department of Biomedical Engineering

Technion – Israel Institute of Technology

[2]The Norman Seiden Multidisciplinary Program for Nanoscience and Nanotechnology, Technion – Israel Institute of Technology, Haifa, Israel
Haifa 32000, Israel

*Email: yshamay@technion.ac.il

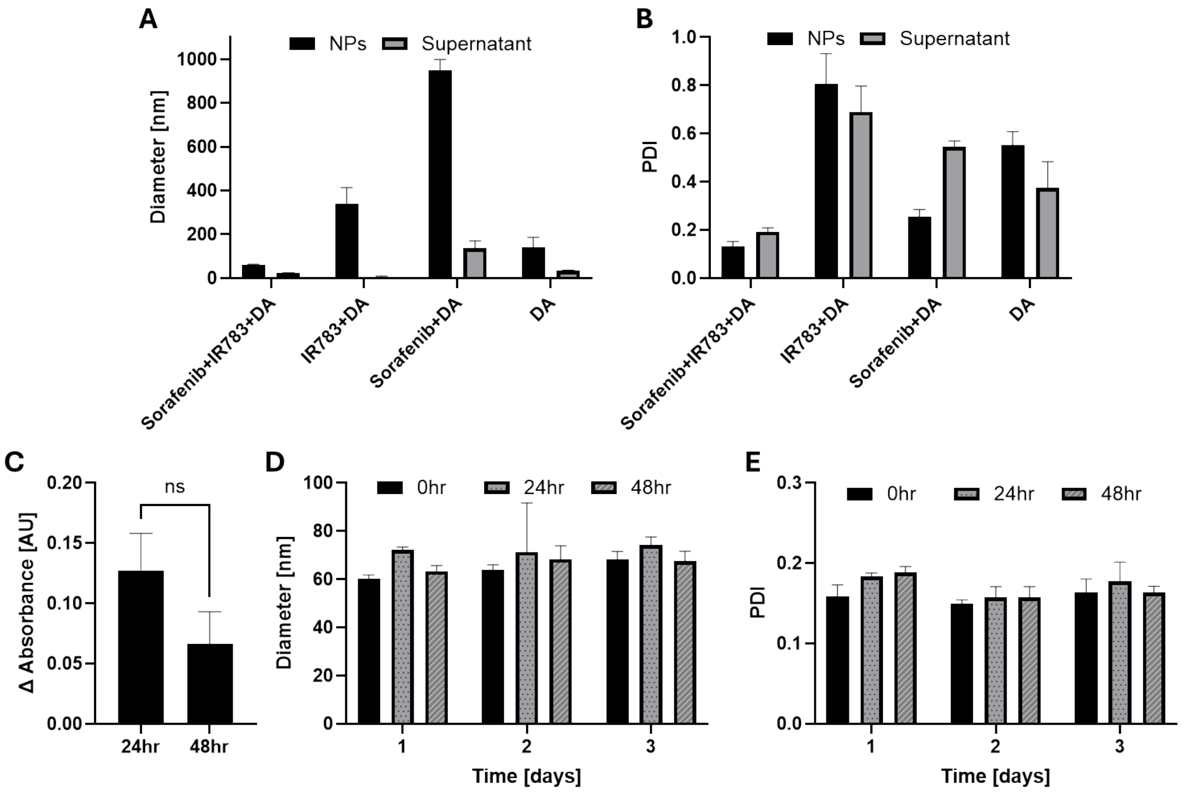

Figure S1- (A-B) Size (A) and PDI (B) of nanoparticles in different component combinations. (C) Difference in absorbance at 490 nm for nanoparticles coated for 24 and 48 hr (p>0.05). (D-E) Size (D) and PDI (E) over three days for non-coated nanoparticles and nanoparticles coated for 24 and 48 hr.

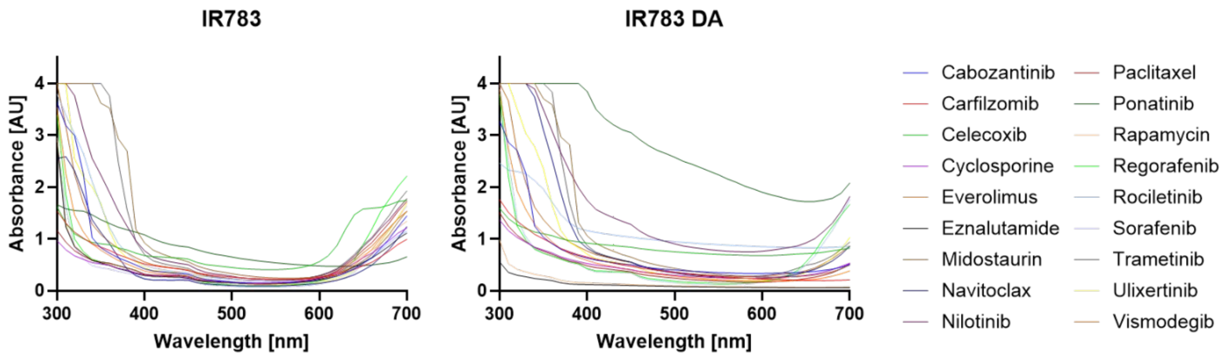

Figure S2- UV-VIS spectra of coated vs. non-coated nanoparticles.

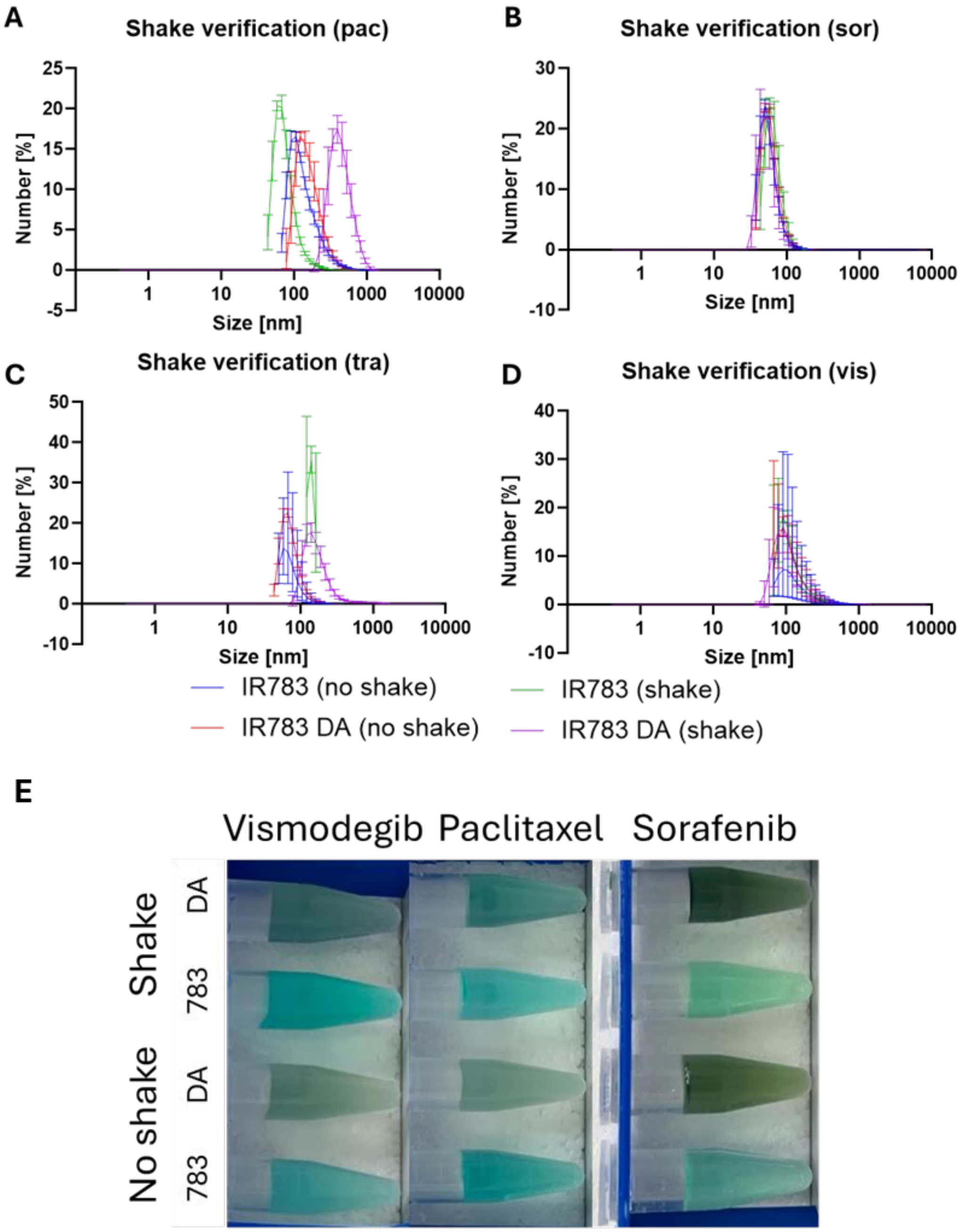

Figure S3- DLS histograms of different drugs with and without shaking during the coating and formation processes. (A) paclitaxel (B) sorafenib (C) trametinib (D) vismodegib. (E) representative photographs of nanoformulations suspensions.

A

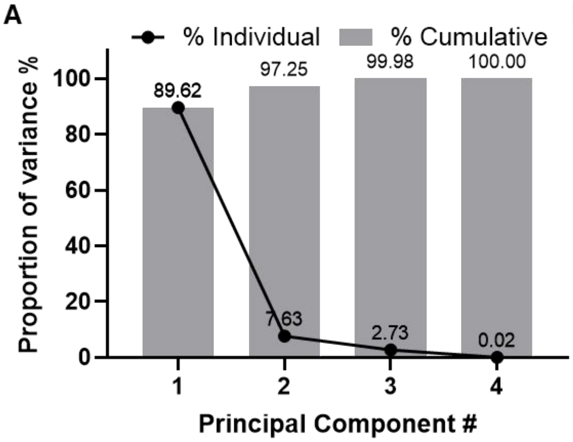

B

Descriptor	PC1	PC2	PC3	PC4
N%	-0.4711	-0.7814	0.4091	0.0103
SdsN	-0.5114	0.4317	0.2179	0.7104
NdsN	-0.5113	0.4278	0.2461	-0.7035
F02[N-N]	-0.5051	-0.1413	-0.8512	-0.0167

Figure S4- PCA data. (A) proportion of variance for the PCs of the analysis. (B) Loadings of the descriptors in the PCs of the analysis.

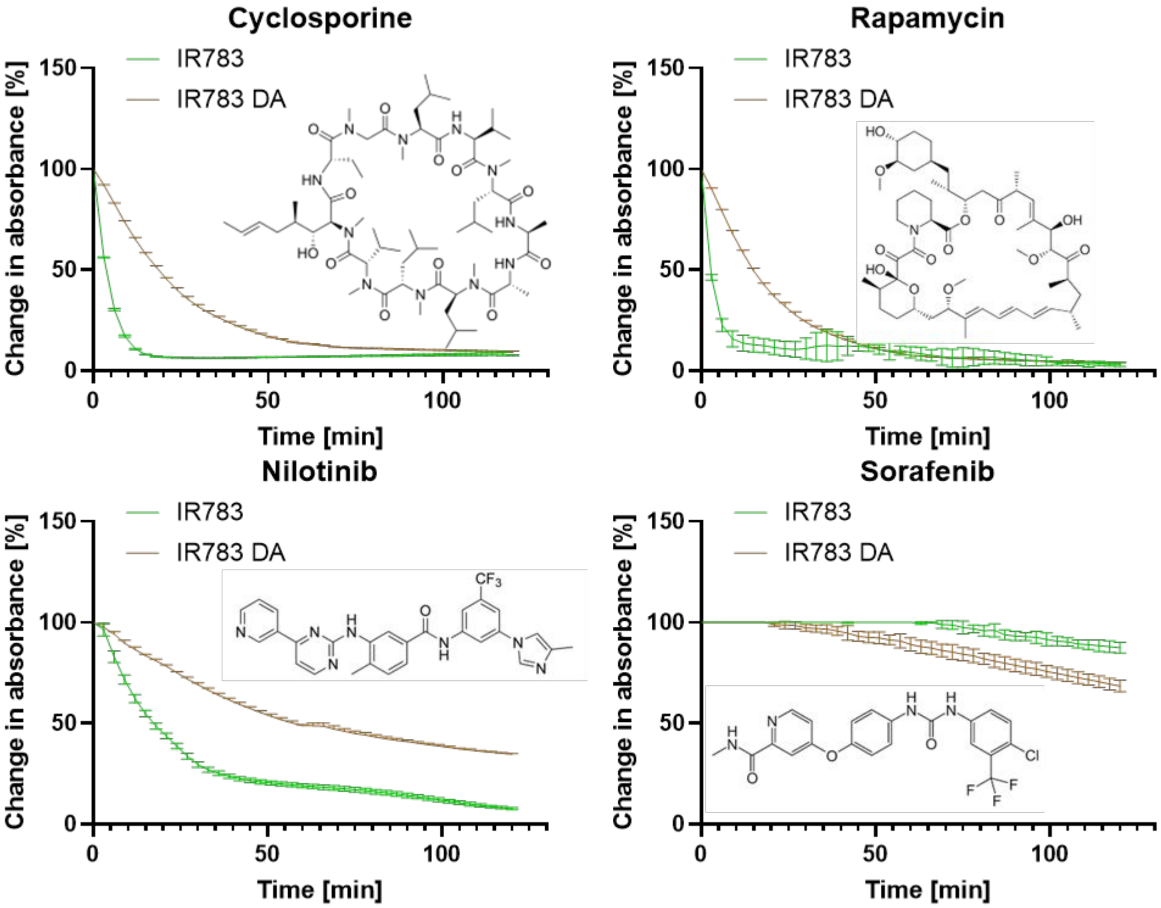

Figure S5- Change in absorbance at 800 nm over 2 hr in H_2O_2 3% solution for coated and non-coated nanoparticles.

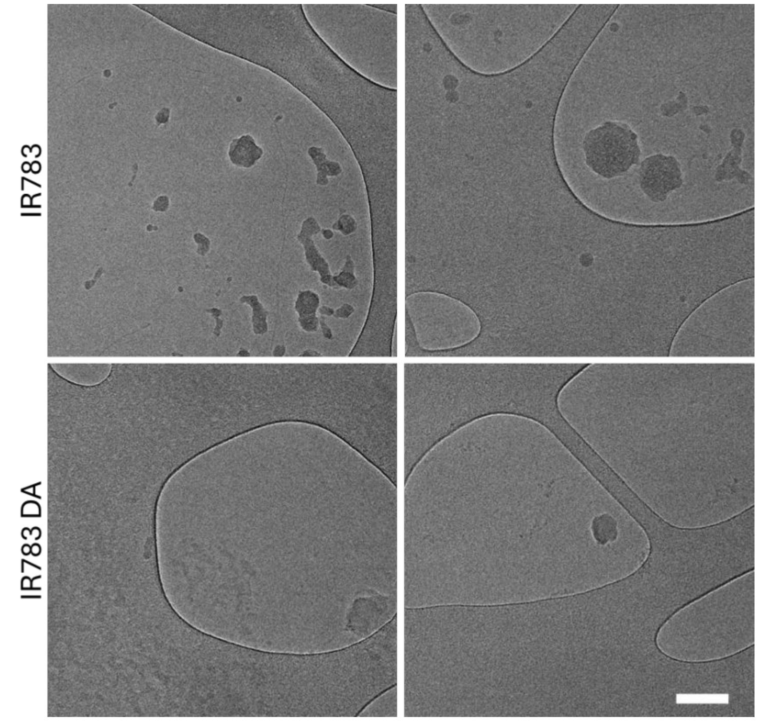

Figure S6- Representative cryoTEM images of non-coated (top) and coated (bottom) trametinib nanoparticles, scale bar = 100 nm.

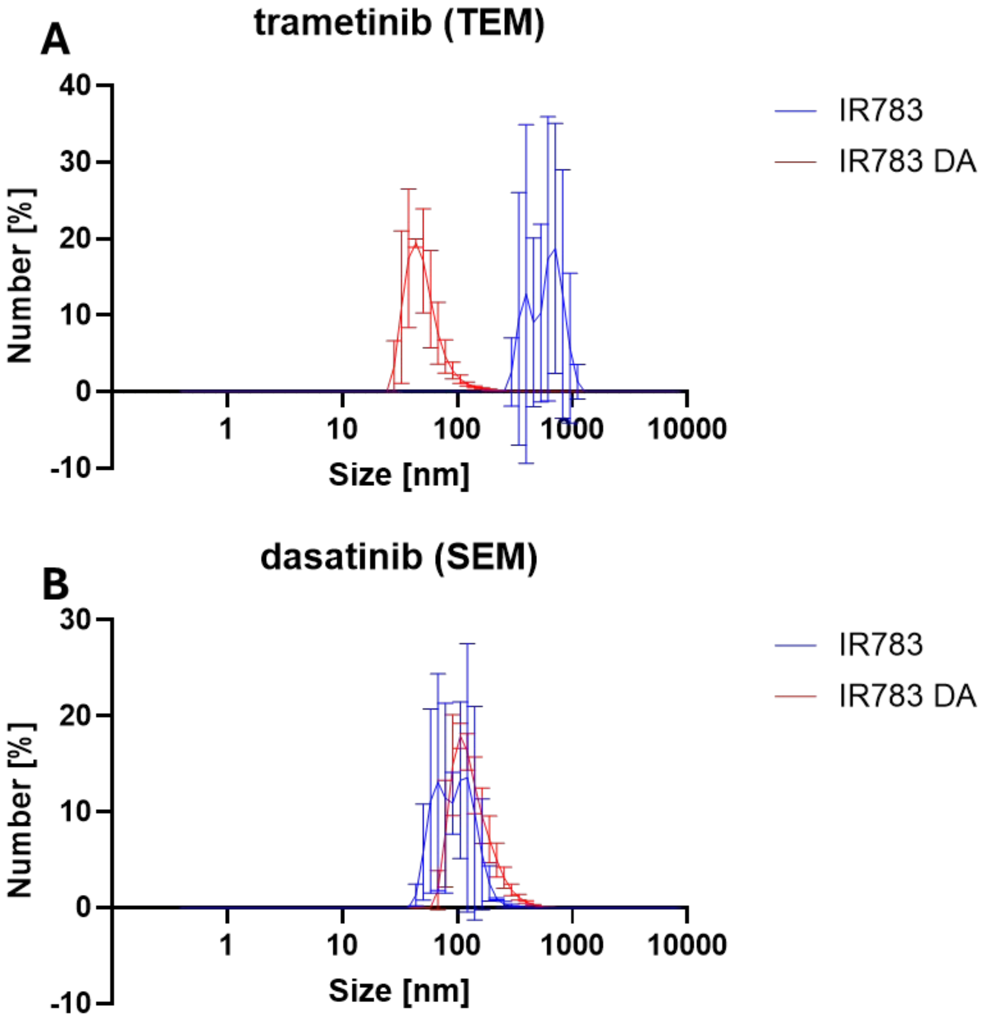

Figure S7- DLS histograms of nanoparticles scanned in cryo-TEM (A) and HR-SEM (B).

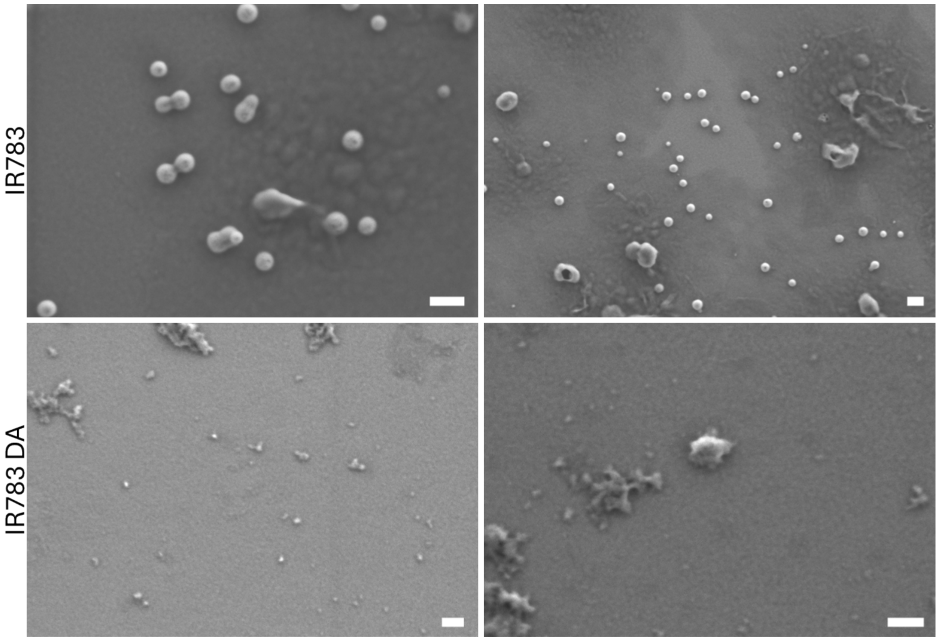

Figure S8- Representative HR-SEM images of non-coated (top) and coated (bottom) dasatinib nanoparticles, scale bars = 200 nm.

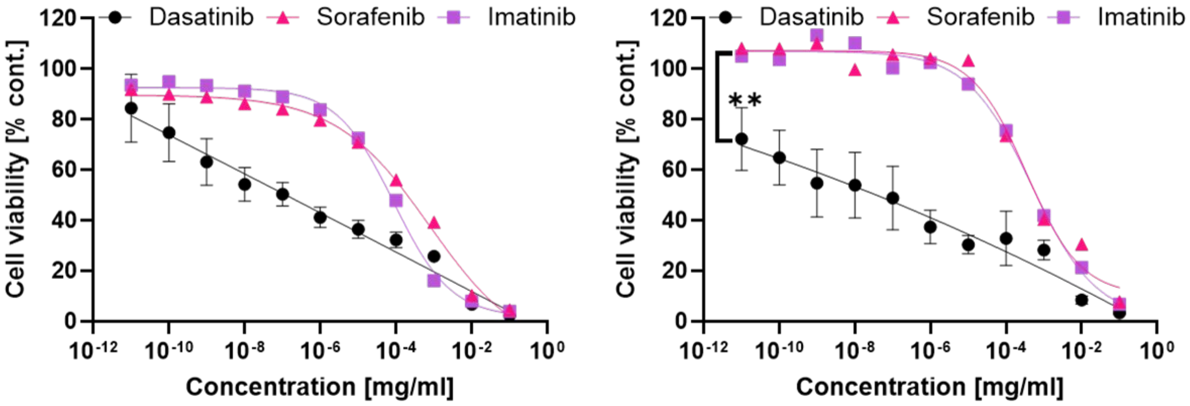

Figure S9- Efficacy of different kinase inhibitors on K7M2 cells in 2D (left) and 3D (right) cell cultures, p=0.0085.

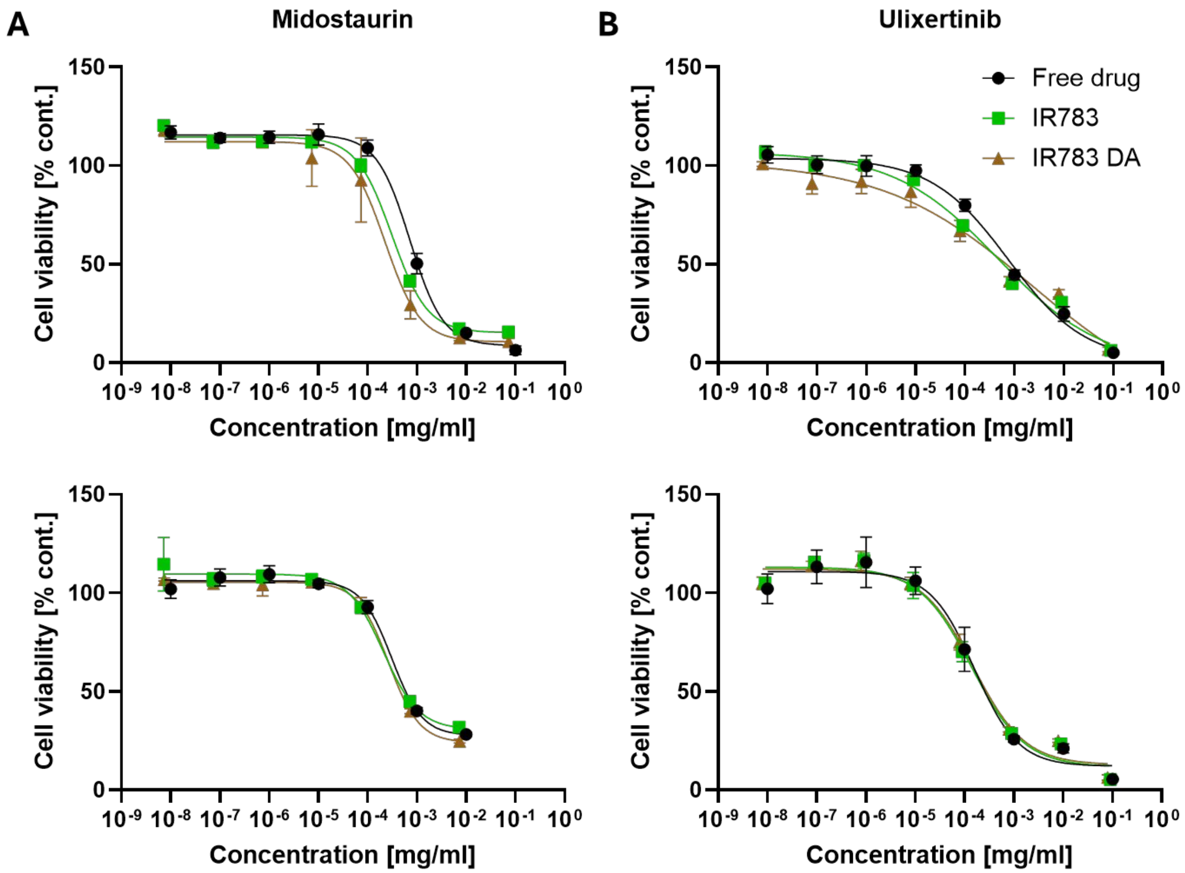

Figure S10- Viability results of HCT116 cells with midostaurin (A) and ulixertinib (B), 2D results are shown at the top panels and 3D results are shown at the bottom panels.

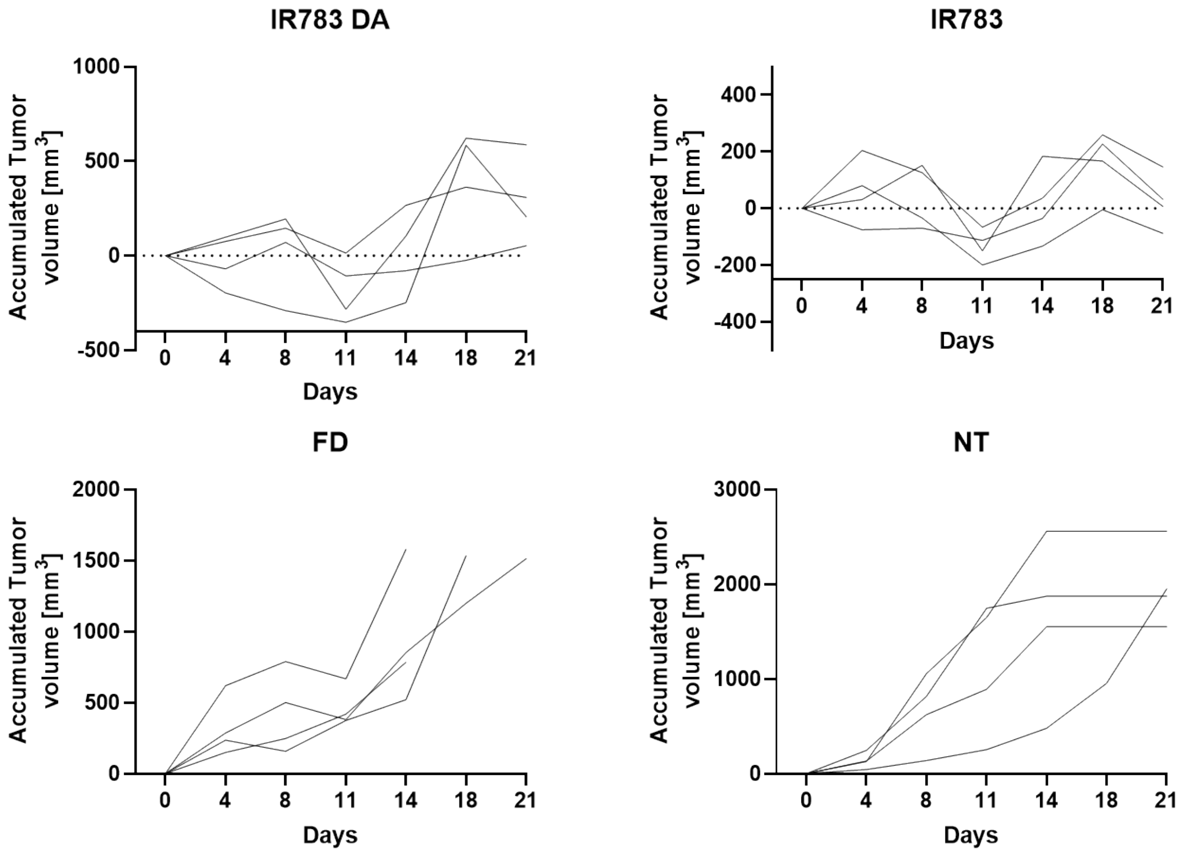

Figure S11- Accumulated tumor volumes for the individual mice in the different treatments.
